# Creation of an Engineered Oxygen-Insensitive L-Glutamate Oxidase for the Application of Electrochemical L-Glutamate Sensors

**DOI:** 10.3390/ijms27062831

**Published:** 2026-03-20

**Authors:** Mika Hatada, Shouhei Takamatsu, Ryutaro Asano, Kazunori Ikebukuro, Wakako Tsugawa, Koji Sode

**Affiliations:** 1 Lampe Joint Department of Biomedical Engineering, The University of North Carolina at Chapel Hill and North Carolina State University, Chapel Hill, NC 27599, USA; mika_hatada@med.unc.edu (M.H.);; 2 Department of Biotechnology and Life Science, Graduate School of Engineering, Tokyo University of Agriculture and Technology, Tokyo 184-8588, Japan; ryutaroa@cc.tuat.ac.jp (R.A.); ikebu@cc.tuat.ac.jp (K.I.); tsugawa@cc.tuat.ac.jp (W.T.)

**Keywords:** L-glutamate oxidase, L-glutamate, neurotransmitter, transient open circuit potential, quasi-direct electron transfer, dye-mediated dehydrogenase activity

## Abstract

L-glutamate (L-Glu) is the primary excitatory neurotransmitter in the mammalian central nervous system. Developing a real-time monitoring system is essential to understanding the onset and progression of related conditions. However, the absence of an L-Glu dehydrogenase that is insensitive to oxygen limits the development of oxygen-independent electrochemical enzymatic sensors. Additionally, the most commonly used L-Glu-specific oxidase requires site-specific proteolytic post-translational modifications in specific host microorganisms, which makes protein engineering difficult. To address these issues, L-Glu oxidase derived from *Streptomyces mobaraensis* (SmEOx), which does not require post-translational modifications, was engineered to function as a dehydrogenase. Residues crucial for the oxidative half reaction with oxygen in SmEOx were identified, and mutagenesis studies were conducted. Mutant SmEOx variants with suppressed oxidase activity and improved dye-mediated dehydrogenase activity compared to the wild-type enzyme were successfully obtained. The ratio of dehydrogenase activity to oxidase activity (Dh/Ox) increased ~2900-fold in mutant M117I and ~6700-fold in mutant M117F/K400N compared to wild-type recombinant SmEOx. The resulting virtually L-Glu dehydrogenases (*v*EDHs) were modified with a redox mediator and evaluated using transient open-circuit potential (OCP)-based L-Glu measurements. As a result, the *v*EDH (M117F/K400N mutant)-immobilized electrode enabled electrochemical L-Glu detection under ambient oxygen without the need for an external electron mediator, unlike the wild-type enzyme. The created *v*EDH, together with the OCP sensor developed using it, paves the way for future development of miniaturized, real-time L-Glu monitoring systems with high temporal and spatial resolution.

## 1. Introduction

L-glutamate (L-Glu) is the primary excitatory neurotransmitter in the mammalian central nervous system (CNS), playing pivotal roles in synaptic transmission, neuroplasticity, and metabolic regulation. Despite its essential physiological functions, L-Glu is a potent neurotoxin when its homeostasis is disrupted. Precise regulation of its concentration is vital, as even minor elevations can trigger excitotoxicity, a pathological process where receptor overstimulation initiates a cascade of neuronal damage and death [1,2,3]. This mechanism is a hallmark of various neurological disorders, ranging from acute insults like traumatic brain injury (TBI) and ischemia to chronic neurodegenerative conditions such as Alzheimer’s and Huntington’s diseases [1,4]. Therefore, real-time monitoring of L-Glu dynamics with high temporal and spatial resolution is critical to understand the onset and progression of these neurological conditions.

In the healthy brain, L-Glu levels are maintained at millimolar (mM) concentrations within neurons, whereas extracellular and cerebrospinal fluid (CSF) levels are strictly regulated within the low micromolar (µM) range [3,5,6,7]. However, during pathological events such as TBI, extracellular concentrations can surge to approximately hundreds of µM, triggering irreversible neuronal damage [4]. Because L-Glu operates within distinct functional compartments such as synaptic and extrasynaptic spaces, these dynamics must be resolved with high spatiotemporal precision. While traditional analytical techniques, such as microdialysis (coupled with HPLC or LC-MS) and magnetic resonance spectroscopy (MRS), provide high sensitivity, they still lack resolution to capture the rapid, localized fluctuations occurring on the millisecond scale within synaptic microdomains.

To overcome these limitations, significant efforts have been made to develop L-Glu biosensors using various biological recognition elements, including enzymes, periplasmic binding proteins (PBPs), and aptamers as highlighted in a recent review [8]. Genetically encoded fluorescent biosensors, such as iGluSnFR, which contains a PBP domain, have recently revolutionized the visualization of glutamate dynamics with high spatial resolution. However, their application is hindered by the necessity for genetic modification, potential interference from other amino acids (iGluSnFR also binds aspartate), and the difficulty of absolute quantification. Electrochemical biosensors, particularly those using FAD-dependent L-Glu oxidase (EOx), remain as the predominant quantitative L-Glu monitoring method due to their high specificity, compatibility with electrochemical detection principles, and ease of miniaturization. Furthermore, several in vivo studies using EOx-immobilized microsensors have been reported [9,10,11,12,13,14,15,16,17,18]. Despite these advancements, most existing EOx-based sensors rely on the principle of first-generation electrochemical enzymatic sensors, in which oxygen serves as the primary electron acceptor for EOx and hydrogen peroxide is detected as the reaction product. Consequently, sensor performance is influenced by the concentration of endogenous oxygen. Additionally, the high overpotential required for hydrogen peroxide oxidation introduces interference effects, which are a common limitation of first-generation sensors. To overcome these issues, eliminating oxygen dependence is desirable. However, no L-Glu-specific dehydrogenase has been identified.

**Figure 1:**
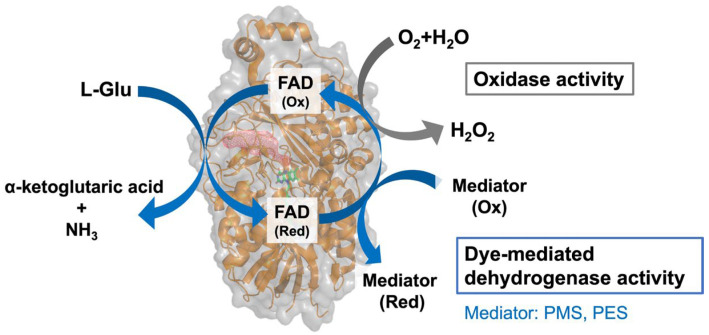
L-glutamate oxidase (EOx) reaction scheme. EOx catalyzes the oxidation of L-Glu to α-ketoglutaric acid and ammonia, utilizing FAD as a cofactor. As an oxidase, which is their native activity, oxygen is used as an electron acceptor and hydrogen peroxide is produced. An artificial electron acceptor (mediator) is used in dye-mediated dehydrogenase activity.

Several EOx enzymes have been identified and cloned from various microorganisms, primarily from the genus *Streptomyces* [19,20,21,22,23,24,25,26,27]. These enzymes catalyze the oxidation of L-Glu to α-ketoglutaric acid and ammonia, using FAD as a cofactor and oxygen as an electron acceptor (Figure 1). Among these enzymes, EOx from *Streptomyces* sp. X-119-6 has been widely used in biosensor development due to its high specific activity [28] and commercial availability as a recombinant enzyme. A major drawback of this enzyme is the requirement for complicated post-translational modifications in a specific host strain: proteolytic cleavage at three sites to generate a mature, active form. Recombinantly prepared enzymes also require this processing step, limiting the flexibility of protein engineering applications. To address this issue, a single-chain variant that does not require protease digestion has been reported [29]. In contrast, an EOx from *Streptomyces mobaraensis* (SmEOx) [26] was reported that naturally does not require proteolytic cleavage, making it a promising candidate for enzyme engineering and biosensor development.

Our research group has been engaged in the engineering of oxidases into dehydrogenases, which are ideal molecules for the application of oxygen-independent electrochemical sensing systems [30]. We have modified the oxidative half-reaction to suppress the reactivity against oxygen by introducing site-specific mutations around the oxygen access site in fructosyl amino acid oxidase, fructosyl peptidyl oxidase, glucose oxidase, cholesterol oxidase, L-lactate oxidase, and glycerol 3-phosphate oxidase [31]. The oxidase activity of these enzymes was successfully decreased while the dye-mediated dehydrogenase activity using artificial electron acceptors was maintained or even improved.

In this study, we attempted to create an FAD-dependent L-Glu dehydrogenase, EDH, by engineering SmEOx [26], which exhibits high catalytic activity without requiring post-translation modifications. First, we predicted the 3D structural model of SmEOx and identified the residues responsible for the oxidative half-reaction with oxygen: Met117 (M117) and Lys400 (K400). This was achieved based on a previous study that reported the structural analysis of L-phenylalanine oxidase (PAO), another FAD-dependent L-amino acid oxidizing enzyme. That study suggested the presence of the oxygen pathway at the *si*-face of flavine of FAD [32]. Next, we carried out strategic mutagenesis studies on those residues in SmEOx by investigating mutants’ oxidase and dye-mediated dehydrogenase activities (Figure 1). Mutant SmEOx variants with suppressed oxidase activity and improved dye-mediated dehydrogenase activity compared to the wild-type (WT) enzyme were successfully obtained. The ratio of dehydrogenase activity to oxidase activity (Dh/Ox) increased ~2900-fold with the mutant M117I and ~6700-fold with the mutant M117F/K400N, compared to WT SmEOx. The selected mutant SmEOxs, which were virtually L-Glu dehydrogenases (*v*EDHs), were evaluated electrochemically based on quasi-DET-dependent transient open circuit potential (OCP) measurements using previously reported mediator modification procedures [33,34]. Finally, we achieved the electrochemical measurement of L-Glu using *v*EDH (SmEOx M117F/K400N double mutant) based on transient OCP, where the signal is measurable even under ambient oxygen and does not require an external electron mediator, which is suitable for the future application of developing miniaturized real-time L-Glu monitoring.

## 2. Results and Discussion

### 2.1. Site-Directed Mutagenesis Study

**Figure 2:**
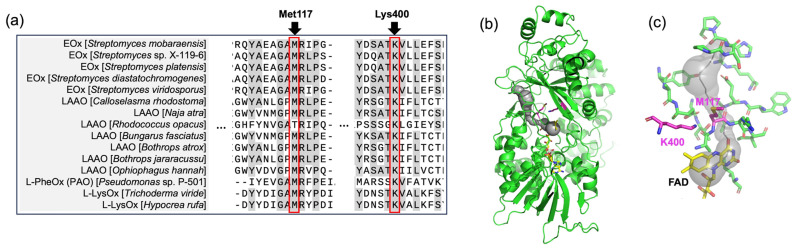
(**a**) Amino acid sequence alignment of L-amino acid oxidizing enzymes including L-glutamate oxidase (EOx), L-amino acid oxidase (LAAO), L-phenylalanine oxidase (L-PheOx, PAO), L-lysine oxidase (L-LysOx). The region around M117 and K400 in SmEOx is shown. (**b**,**c**) Model structure of SmEOx predicted by AlphaFold2 after energy minimization using GROMACS. The cavity for oxygen access predicted using CAVER is shown with the gray sphere. Overall structure (**b**) and the residues located around the oxygen tunnel including M117 and K400 (**c**) are shown.

Ida et al. investigated the oxygen channel in PAO from *Pseudomonas* sp. P-501 based on the experimental structure [32]. The channels in the crystal structure of PAO were predicted using the program CAVER and the widest channel was found at *si*-face of the flavin ring. They focused on the gate residues, M142 and K478, which are located at the bottleneck of the tunnel, and performed site-directed mutagenesis studies on them (M142A and K478A). Both M142A and K478A showed significantly lower catalytic activity, and M142A had elevated apparent *K*_m_ value against oxygen compared to the WT. These results demonstrated M142 and K478 are crucial residues for the oxidative half-reaction with oxygen of PAO. Significantly, these two residues are widely conserved among L-amino acid oxidizing enzymes including SmEOx (Figure 2a). Moreover, when comparing the crystal structure of PAO (PDB: 2YR4) with the model structure of SmEOx, these residues are aligned at equivalent positions. Therefore, we decided to focus on the two residues, M117 and K400 in SmEOx, corresponding to M142 and K478 in PAO, as the target for the mutagenesis study to alter the oxidase activity of SmEOx. The channel from FAD to outside of the protein was predicted by CAVER software using the model structure of SmEOx WT (Figure 2b,c). A channel passing near M117 and K400 was predicted at the same position as seen in PAO at the *si*-face of flavin ring (Figure 2b). This observation also supports the idea that these residues are the appropriate candidates for the mutagenesis study.

We first conducted saturation mutagenesis studies on both M117 and K400 residues, respectively. The results of activity assays of each crude enzyme sample are shown in Supplementary Information (Figure S1). All the single mutants showed suppressed oxidase activity, and almost of them showed increased dehydrogenase activity. These observations demonstrated that M117 and K400 are crucial residues for oxygen binding in SmEOx, too. M117F, M117I, M117E, M117Q, K400M, K400I, K400L and K400N showed the highest increase in the dye-mediated dehydrogenase activity among the mutations to each residue (M117 or K400), while their oxidase activity compared to the WT was decreased. Therefore, a double mutant of the combination of M117F, I, E, Q and K400M, I, L, N were constructed and evaluated. The activity assay results are also shown in Figure S1. All the double mutants showed a significant decrease in oxidase activity. Among the double mutants, M117I/K400I, M117F/K400I and M117F/K400N showed the highest increase in dehydrogenase activity compared to the WT.

**Figure 3:**
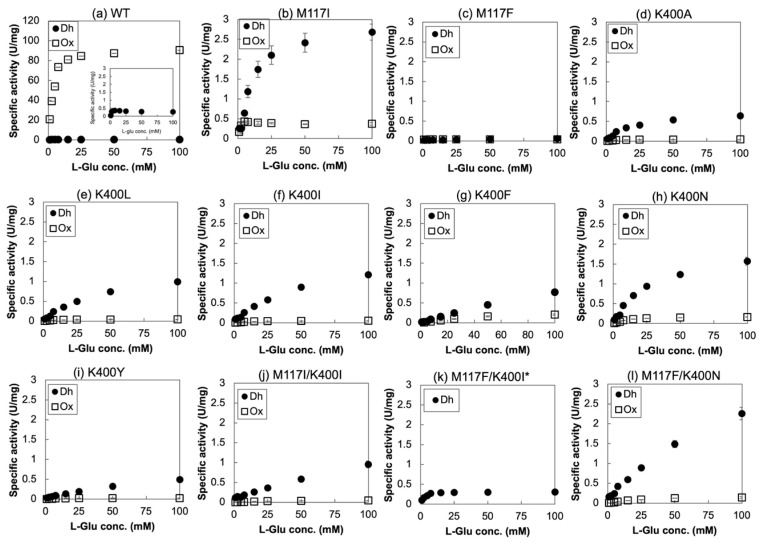
Enzymatic activity of purified SmEOx WT and each mutant. Oxidase activity (Ox) and dye-mediated dehydrogenase activity (Dh) were measured against various concentrations of L-Glu. (**a**) WT, (**b**) M117I, (**c**) M117F, (**d**) K400A, (**e**) K400L, (**f**) K400I, (**g**) K400F, (**h**) K400N, (**i**) K400Y, (**j**) M117I/K400I, (**k**) M117F/K400I, (**l**) M117F/K400N. * Oxidase activity was not observed for M117F/K400I mutant.

Among all the mutants, single mutants of M117I, M117F, K400A, K400L, K400I, K400F, K400N and K400Y, and double mutants of M117I/K400I, M117F/K400I and M117F/K400N were selected for further characterization and subjected to purification. SDS-PAGE analysis of the purified samples is shown in Figure S2. Purification with Ni-affinity chromatography led to high-purity samples for all the prepared mutants. Purified enzymes including the WT were then evaluated for their oxidase and dye-mediated dehydrogenase activity. Figure 3a–l shows the results of both oxidase and dye-mediated dehydrogenase activity toward varied concentrations of L-Glu. Based on these results, Hanes–Woolf plots were prepared and *K*_m_ and *V*_max_ values for both activities were calculated and are summarized in Table 1.

All the characterized mutants had significantly low oxidase activity compared to the WT (more than 200 times lower *V*_max_ compared to the WT). As for the dehydrogenase activity, M117I and M117F/K400N mutants showed the highest activity with a *V*_max_ of around 3 U/mg (more than 10 times higher than the WT). All the single mutants on K400 (K400A, L, I, F, N, or Y) and one double mutant M117I/K400I showed a moderate increase with 2.5–8.6 times higher dehydrogenase activity than the WT. M117F and M117F/K400I did not show any improvement. Interestingly, M117F and M117F/K400I showed a near-complete loss of both oxidase and dehydrogenase activities, whereas M117F/K400N displayed the greatest dehydrogenase activity with depleted oxidase activity. M117F alone or in combination with K400I appeared to have significantly low activity, possibly due to steric hindrance caused by the substitution of Met with bulky Phe, which likely resulted in poor packing with surrounding residues; however, when paired with K400N, M117F may have been positioned favorably, enabling efficient blockage of the oxygen channel. Significantly, the ratio of dehydrogenase activity against oxidase activity (Dh/Ox) was improved by ~2900 times with the M117I mutant, and by ~6700 times with the M117F/K400N mutant, whose dehydrogenase activities were highest among the mutants.

In this study we focused exclusively on altering the enzyme’s reactivity toward oxygen without affecting interactions with either the substrate or the mediator. In SmEOx, both the substrate and mediator appear to approach the FAD cofactor from the *re*-face, while oxygen approaches from the opposite *si*-face. We specifically targeted the oxygen pathway on the *si*-face by introducing mutations at residues M117 and K400. As expected, these substitutions did not markedly affect mediator affinity because the apparent *K*_m_ values for PMS (*K*_m *PMS*_) remained comparable between the WT and the K400F mutant, which showed the highest *K*_m_ for L-Glu among the mutants in terms of dehydrogenase activity (WT: *K*_m *PMS*_ = 0.15 mM, K400F: *K*_m *PMS*_ = 0.21 mM, Supplementary Information Figure S3).

These results demonstrated that we successfully altered the oxygen reactivity and created SmEOx mutants, which have repressed oxidase activity and significantly improved dehydrogenase activity, and are virtually L-Glu dehydrogenases (*v*EDHs). They are suitable for the application of electrochemical sensor development.

### 2.2. Electrochemical Evaluation of vEDHs

Electrochemical evaluation was performed based on OCP measurement for M117I and M117F/K400N, which showed the greatest improvement in dehydrogenase activity, as well as for M117F/K400I, which did not enhance dehydrogenase activity but lost oxidase activity, in addition to the WT. The enzymes were immobilized on the gold disk electrode (GDE) via a self-assembled monolayer (SAM) harboring an NTA group, which binds with the His-tag on the enzymes. Following immobilization of the enzyme onto the electrode, the mediator arPES was modified on the enzyme surface via amine coupling between the succinimide group in arPES and the amino groups of the surface-exposed Lys residues of SmEOxs. The successful immobilization of SmEOxs and its modification by arPES was confirmed by CV measurement (Figure S5). All electrodes showed redox peak currents at the potential corresponding to the redox potential of PES, demonstrating that all the SmEOx variants (WT, M117I, M117F/K400I and M117F/K400N) were immobilized on the gold electrodes, modified with arPES, and that arPES could react with the electrode.

**Figure 4:**
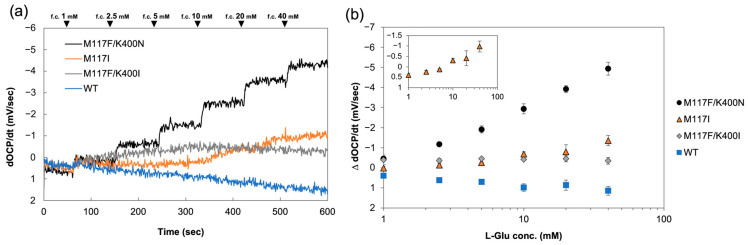
Transient OCP measurement using arPES-modified SmEOx WT, M117I, M117F/K400I, and M117F/K400N mutants. (**a**) Time-dependent transient OCP (dOCP/dt) change. (**b**) Calibration curve of ∆ dOCP/dt against L-Glu concentration.

**Figure 5:**
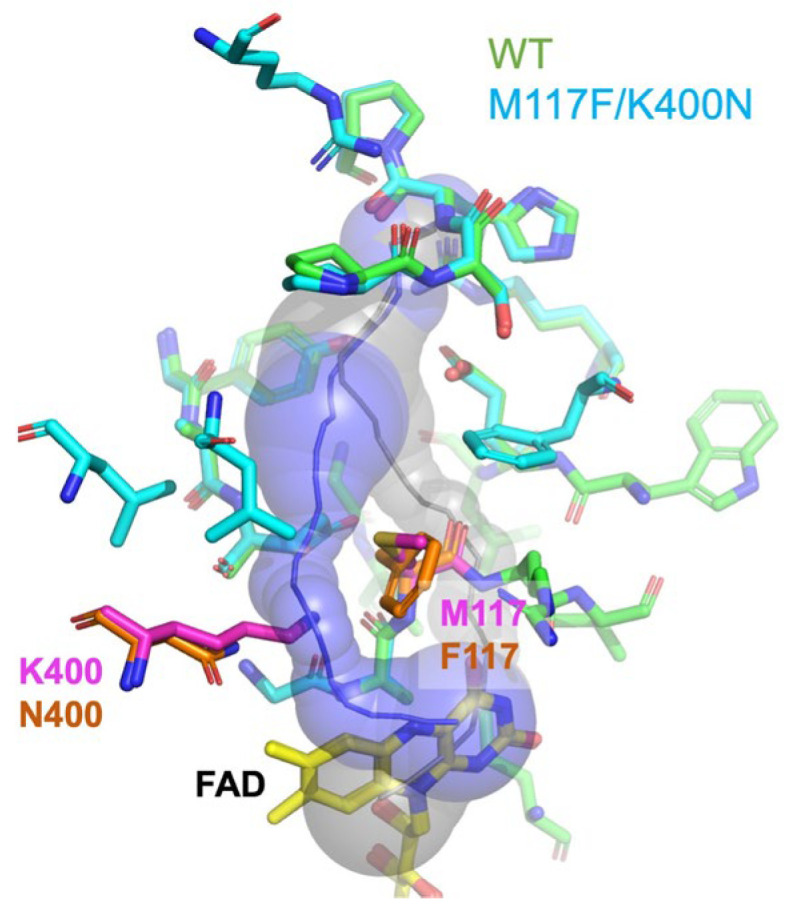
Comparison of the oxygen tunnel predicted in WT SmEOx and M117F/K400N mutant. Predicted tunnels (gray sphere: WT, blue sphere: M117F/K400N) and residues around predicted tunnels (light green: WT, light blue: M117F/K400N) in WT and M117F/K400N SmEOx are superimposed. M117 and K400 residues in WT SmEOx are shown in magenta and the corresponding mutated residues F117 and N400 in the M117F/K400N mutant are shown in orange.

Then, L-Glu dependent electrochemical signals were evaluated by quasi-DET type OCP measurement under ambient atmosphere, and the transient OCP (dOCP/dt) was analyzed according to our previous studies [33,34]. In the OCP measurement procedure for quasi-DET enzyme sensors, a potential of +100 mV vs. Ag/AgCl is first applied briefly to fully oxidize the arPES mediator covalently bound to the enzyme, resetting the electrode to a reproducible oxidized state, then the OCP signal is recorded, and this cycle is repeated. Addition of substrate (L-Glu) triggers the enzyme reaction, where the substrate is oxidized and the FAD cofactor is reduced. Electrons then transfer from reduced FAD to arPES, increasing the reduced mediator ratio as the reaction proceeds. This shifts the electrode OCP based on the changing oxidized/reduced mediator ratio following the Nernst equation, yielding a concentration-dependent potential decrease. The derivative dOCP/dt captures the initial rate of this change, directly correlating with the enzyme’s substrate oxidation rate and rising reduced mediator fraction. The representative raw data of the time dependent OCP change is shown in Figure S6a–e. The dOCP/dt, calculated from measured OCP with each SmEOx immobilized electrode, is shown in Figure 4. Among the tested SmEOxs, only the M117F/K400N mutant immobilized electrode showed a clear L-Glu-dependent response with transient OCP. The change in the dOCP/dt against the blank (∆ dOCP/dt) was plotted against L-Glu concentration to make calibration curves (Figure 4b). While immobilized electrodes with the WT, M117I, or M117F/K400I mutants showed no quasi-DET-dependent signal change or only a slight change by the addition of L-Glu, M117F/K400N showed increasing quasi-DET-dependent transient OCP over the tested L-Glu concentration (1–40 mM). Since this experiment was conducted under ambient air conditions, the primary electron acceptor of WT SmEOx is oxygen. Therefore, the WT immobilized electrode did not exhibit a signal change based on quasi-DET. Instead, the WT electrode showed a slight signal change in the opposite direction compared to M117F/K400N, which might be attributed to the hydrogen peroxide produced during the reaction. M117F/K400I completely lost oxidase activity and exhibited markedly low dehydrogenase activity. Therefore, the absence of an electrochemical signal was likely due to its overall low enzymatic activity. Additionally, the fact that the M117F/K400I-immobilized electrode did not show any OCP signal change effectively serves as a negative control, indicating that L-Glu itself does not generate OCP change. The M117I electrode showed only a very slight change in signal at higher L-Glu concentrations (>10 mM) (Figure 4b, inset). Although M117I exhibited a marked improvement in dehydrogenase activity, its oxidase activity remained relatively high compared with the other variants (Figure 3). In fact, M117I showed higher residual oxidase activity than M117F/K400N, particularly at lower L-Glu concentrations (Figure S4), while both mutants exhibited similar dehydrogenase activity values (Table 1). As a result, the Dh/Ox ratio was 2.3-fold higher for M117F/K400N at *V*_max_ (Table 1). At low L-Glu concentrations (1 mM), which are critical for physiological measurements, M117F/K400N showed a Dh/Ox ratio of 61, whereas M117I shows only 1.6 (a 38-fold higher Dh/Ox ratio for M117F/K400N), demonstrating a critical difference between them. This residual oxidase activity in M117I likely reduced the efficiency of electron transfer to the mediator, causing electron leakage to oxygen and preventing the generation of an efficient quasi-DET-dependent signal change. In contrast, M117F/K400N not only exhibited a remarkable increase in dehydrogenase activity but also showed a pronounced decrease in oxidase activity (high Dh/Ox ratio). This reduction in electron transfer to oxygen likely improved the efficiency of electron transfer to the mediator, resulting in a clear quasi-DET derived signal change. Structural analysis of PAO suggests that K478 plays a critical role in oxygen recognition by stabilizing molecular oxygen near FAD through its positive charge [32]. The corresponding residue in SmEOx is K400. Substitution with Asn (K400N) removes this positive charge, eliminating stabilization and preventing oxygen from efficiently accessing FAD. In addition, the M117 to Phe substitution introduces steric hindrance near the channel, further restricting oxygen entry. Together, these mutations likely block oxygen access to FAD, resulting in the loss of oxidase activity and enabling efficient quasi-DET. Consistent with this interpretation, CAVER analysis revealed that the canonical oxygen pathway predicted for the WT enzyme was absent in M117F/K400N, and an alternative route was formed (Figure 5). Although a new pathway was predicted in the mutant, the absence of K400 likely prevents effective binding and stabilization of oxygen in proximity to FAD. This observation strongly supports the hypothesis that these substitutions remodel both the internal oxygen access pathway and the binding site, effectively limiting oxygen availability for the oxidative half-reaction.

The apparent *K*_m_ value calculated from the transient OCP result of M117F/K400N was 12 mM, which is lower than the *K*_m_ value obtained from the homogeneous dehydrogenase activity assay using PMS as the electron acceptor (63 mM). This difference may be explained by the fact that, in the electrochemical system, only arPES mediators located close enough to the FAD cofactor can effectively accept electrons, even though several arPES molecules can attach to the enzyme surface due to the presence of multiple lysine residues. Indeed, an average of 9.6 arPES molecules were attached per SmEOx enzyme (~46% modification ratio relative to 21 surface-exposed Lys residues), as quantified spectroscopically using arPES-modified SmEOx prepared by incubating the enzyme with arPES in homogeneous solution (Supplementary Information Figure S7). This limitation in available mediators for the electron transfer from FAD restricts the oxidative half-reaction and leads to saturation at lower substrate concentrations compared to the homogeneous system, where electron acceptors are abundant.

The limit of detection (LOD), calculated using the standard method of three times the standard deviation of the blank (3σ), was 0.25 mM. Although L-Glu transiently spikes to millimolar levels during neurotransmission, its basal extracellular concentration and cerebrospinal fluid (CSF) levels are typically in the micromolar range. Therefore, a detection range in the micromolar level is ideal for physiological monitoring. The relatively high LOD (0.25 mM) and apparent *K*_m_ (12 mM) observed for the transient OCP measurement using the M117F/K400N mutant may be attributed to SmEOx’s lower reactivity toward artificial electron acceptors and suboptimal electron transfer efficiency between the FAD cofactor and the surface-bound arPES mediator. WT SmEOx exhibits high oxidase activity (*V*_max_ = 93 U/mg, *K*_m_ = 3.0 mM, *V*_max_/*K*_m_ = 31 U·mg^−1^·mM^−1^) when oxygen serves as the primary electron acceptor, reflecting the inherent function of SmEOx. This *K*_m_ value is comparable to that of the most widely used enzyme for existing electrochemical L-Glu monitoring, the commercially available EOx from *Streptomyces* sp. X-119-6 (*K*_m_ = 0.23 mM). Therefore, the ability of SmEOx to recognize and oxidize L-Glu should be sufficient to detect physiological L-Glu concentrations. In contrast, the dehydrogenase mutant M117F/K400N shows low dye-mediated activity (*V*_max_ = 3.5 U/mg, *K*_m_ = 63 mM, *V*_max_/*K*_m_ = 0.056 U·mg^−1^·mM^−1^), even though this represents an improvement compared to the WT enzyme in terms of mediator-based electron transfer. This lower activity is likely due to poor mediator interaction. However, this limitation is inherent not only to SmEOx, but also to currently available EOxs, which generally exhibit low activity when combined with artificial or synthetic electron acceptors for the oxidative half-reaction compared to their primary and native electron acceptor, oxygen. This challenge is precisely what this study aims to address by repressing oxidase activity and thereby enhancing the enzyme’s ability to utilize artificial or synthetic electron acceptors for the oxidative half-reaction. Our results clearly demonstrate that the ability to use a synthetic electron acceptor was improved by reducing electron transfer to oxygen, which otherwise competes with the synthetic acceptor under ambient oxygen conditions. These findings indicate that our engineering strategies represent a promising pathway toward realizing a non-oxygen-dependent enzymatic sensor for Glu detection.

To understand the reason of the low dye-mediated dehydrogenase activity after PES modification, and also to find a way to improve the activity, we investigated possible positions of the surface-exposed Lys residues where PES would be modified. The shortest distance from the N5 atom of FAD to PES was evaluated as 29.9 Å by hypothesizing that Lys (K171), which exists in the closest position to FAD, is modified by PES (Supplementary Information, Figure S8). This distance is relatively long for efficient electron transfer and is close to the maximum center-to-center distance reported for single-step electron tunneling through proteins, which can be no more than 20 Å [35,36]. This long FAD-to-PES distance likely limited the electron transfer efficiency between the FAD cofactor and surface-bound arPES mediator, resulting in reduced sensitivity and an elevated LOD. The electron transfer between the FAD and the surface-bound arPES is expected to be improved by introducing a Lys residue closer to FAD as the site for arPES modification. We previously demonstrated that this strategy enhances electron transfer in other enzymes, including glucose oxidase (GOx), lactate oxidase (LOx), and fructosyl peptide oxidase (FPOx), by enabling closer arPES binding to their cofactors [37]. Further engineering of SmEOx through Lys mutations is expected to enhance FAD-to-mediator electron transfer, improve sensitivity, and achieve physiologically relevant detection limits in future applications.

SmEOx has been reported to exhibit high specificity for L-Glu, with negligible reactivity toward other amino acids, including L-Asp and L-Gln. SmEOx also remained stable after 1 h of incubation at 35 °C [26]. These results suggest that a sensor employing SmEOx or its dehydrogenase mutant would exhibit sufficient L-Glu selectivity and short-term stability (several hours but not days), which is adequate for neuroscientific investigations. Our previous study demonstrated that a D-Ser sensor based on arPES-modified D-amino acid oxidase (DAAO) using OCP sensing functioned successfully in artificial CSF (aCSF). These results demonstrated the feasibility of arPES and arPES-modified enzymes in complex matrices [33].

Recently, Han et al. reported the site-directed modification of an osmium complex (Os) mediator onto EOx from *Streptomyces* sp. X-119-6 by introducing Cys residues for thiol-reactive Os conjugation [38]. Selective anchoring through Cys introduction successfully positioned the Os-mediator close to FAD and resulted in higher electron-transfer efficiency compared with the WT enzyme. However, because the oxygen reactivity of the enzyme remained unchanged, their electrochemical evaluation required anaerobic conditions, where oxygen was absent and the mediator served as the sole electron acceptor for the Os-mediated signal. In contrast, our engineered SmEOx enables mediator-based L-Glu detection under ambient oxygen. Furthermore, their EOx from *Streptomyces* sp. X-119-6 requires proteolytic maturation after expression to achieve full activity. The SmEOx used in this study does not require such processing, making it more amenable to protein engineering. To the best of our knowledge, this is the first study to report an engineered EOx with minimal reactivity toward oxygen, a *v*EDH, that enables mediator-based electrochemical detection of L-Glu under ambient oxygen conditions. Furthermore, we demonstrated that the *v*EDH we developed allows OCP-based L-Glu measurement using a mediator-modified enzyme under ambient oxygen condition. An additional advantage of using OCP as the sensing principle is that the signal is independent of electrode area, unlike conventional amperometric methods, which dominate existing studies that measure hydrogen peroxide produced by the oxidase activity of EOx. Therefore, now that dehydrogenase conversion has been achieved, further optimization of electron transfer efficiency with the mediator will enable the development of miniaturized sensors suitable for neurotransmitter monitoring.

## 3. Materials and Methods

### 3.1. Chemicals and Materials

L-glutamate (L-Glu) was purchased from Merck KGaA (Darmstadt, Germany). 4-aminoantipyrine (4-AA), KH_2_PO_4_, K_2_HPO_4_, (NH_4_)_2_SO_4_, Na_2_HPO_4_, MgSO_4_, NaCl, and ethanol were obtained from Kanto Kagaku (Tokyo, Japan). Imidazole, glycerol, lactose monohydrate, and kanamycin sulfate were purchased from FUJIFILM Wako Pure Chemical (Osaka, Japan). LB broth and ultrafiltration filters (Amicon Ultra-15 Centrifugal Filter Unit 30-K) were purchased from Merck KGaA (Darmstadt, Germany). 2,6-Dichloroindophenol (DCIP), Phenazine methosulfate (PMS), N-Ethyl-N-(2-hydroxy-3-sulfopropyl)-3-methylaniline sodium salt (TOOS), 3,3′-Dithiobis[N-(5-amino-5-carboxypentyl) propionamide-N′,N′-diacetic acid] dihydrochloride (Dithiobis (C2-NTA)) and 1-[3(succinimidyloxycarbonyl)propoxy]-5-ethylphenazinium trifluoromethanesulfonate (amine-reactive phenazine ethosulfate; arPES) were purchased from Dojindo Laboratories (Kumamoto, Japan). Peroxidase was purchased from Amano Enzyme (Gifu, Japan). All chemicals were of reagent grade. 1 mL HisTrap HP columns were purchased from Cytiva (Marlborough, MA, USA). Gold (Au) disk electrodes (GDE; diameter of 3 mm, surface area of 7 mm^2^), platinum (Pt) wire, and a silver/silver chloride (Ag/AgCl) reference electrode were purchased from BAS Inc. (Tokyo, Japan). Potentiostats, SP-50, SP-150, and VSP from Bio-Logic (Claix, France) were used for the electrochemical experiments.

### 3.2. Structure Modeling and Channel Prediction

The model structure of SmEOx WT and M117F/K400N were predicted using AlphaFold2 [39], and were subjected to energy minimization using GROMACS [40]. The channel from the N5 atom of FAD to the outside of the protein was predicted using CAVER 3.0.3 [41]. These structures were visualized using PyMOL Molecular Graphics System, Version 3.0 Schrödinger, LLC (New York, NY, USA).

### 3.3. Preparation of Recombinant SmEOx Wild Type and Mutants

A gene of EOx from *Streptomyces mobaraensis* (SmEOx) was synthesized (Integrated DNA Technologies, Coralville, IA, USA) and inserted into the multiple-cloning site of pET28a (+) vector (Merck KGaA, Darmstadt, Germany). Site-directed mutagenesis was performed via QuikChange to construct the expression vectors for the mutant SmEOxs.

For recombinant production, *E. coli* BL21(DE3)-LOBSTR strain (Kerafast Inc., Boston, MA, USA) was transformed with the plasmids containing SmEOx WT or mutants, and cultured aerobically in 100 mL of ZYP-5052 medium (0.5% glycerol, 0.05% glucose, 0.2% lactose, 50 mM KH_2_PO_4_, 25 mM (NH_4_)_2_SO_4_, 50 mM Na_2_HPO_4_, and 1 mM MgSO_4_) containing 50 µg/mL kanamycin at 30 °C for 24 h. Cells were harvested by centrifugation, resuspended in 20 mM potassium phosphate buffer (PPB) pH 6.0 containing 500 mM NaCl, and disrupted by a French press. After centrifugation (10,000× *g*, 4 °C, 10 min) and ultracentrifugation (130,000× *g*, 4 °C, 60 min), the supernatant was used as the soluble fraction and subjected to the Ni^2+^ affinity column chromatography using an ÄKTA pure system and a 1 mL HisTrap HP column (Cytiva, Marlborough, MA, USA). The eluted fractions containing SmEOx (confirmed by the monitored absorbance at 450 nm derived from FAD and SDS-PAGE) were concentrated and buffer-exchanged to 20 mM PPB pH 6.0 using Amicon 30-K filters (Merck KGaA, Darmstadt, Germany), and stored at −80 °C until use.

### 3.4. Enzyme Assays

An oxidase activity assay was conducted in 100 mM PPB pH 6.0 containing 1.5 mM 4-AA, 1.5 mM TOOS, 2.0 U/mL peroxidase (POD) as final concentrations, and various concentrations of L-Glu by monitoring the absorbance change at 555 nm derived from the formation of the quinoneimine dye. One unit of oxidase activity was defined as the amount of enzyme catalyzing the production of 1 μmol H_2_O_2_ per minute. Dye-mediated dehydrogenase activity was measured in the presence of 6 mM PMS, 0.06 mM DCIP and various concentrations of L-Glu in 100 mM PPB pH 6.0 by monitoring the absorbance change at 600 nm, which is associated with the reduction of DCIP. One unit of dehydrogenase activity was defined as the amount of enzyme necessary to catalyze the reduction of 1 μmol of DCIP per minute. Both oxidase and dehydrogenase activity assays were conducted under ambient conditions at room temperature. Protein concentrations were determined by Bradford assay (Bio-Rad Laboratories, Hercules, CA, USA).

### 3.5. Electrochemical Evaluation of SmEOx Wild Type and Mutants

To prepare the PES-modified EOx immobilized electrodes, first, the cleaned GDE was incubated in 50 µM C_2_-NTA dissolved in ethanol overnight at 25 °C, followed by an incubation in 40 mM NiCl_2_ solution for 2 h. The prepared Ni^2+^/NTA-SAM electrodes were then incubated in 1.0 mg/mL SmEOx WT or mutants at 4 °C overnight. After rinsing with 20 mM PPB pH 6.0, the SmEOx immobilized electrodes were incubated in 1.66 mM arPES solution (20 mM tricine buffer pH 6.0) at 4 °C overnight to modify PES on the surface of the enzymes. The electrodes were rinsed with 20 mM PPB pH 6.0 and stored at 4 °C until use.

OCP measurement was performed in 10 mL of 100 mM PPB pH 6.0 by alternating the oxidation potential application at 100 mV vs. Ag/AgCl for 0.1 s and recording OCP for 1.0 s repeatedly while adding various concentrations of L-Glu (f.c. 0, 1, 2.5, 5, 10, 20, 40 mM) into the test solution. An Ag/AgCl electrode and Pt wire were used as reference and counter electrode, respectively.

### 3.6. Generative AI Usage

Microsoft Copilot “https://copilot.microsoft.com (accessed on 11 December 2025)”, DeepL “https://www.deepl.com/en/translator (accessed on 23 December 2025)” and Perplexity Pro “https://www.perplexity.ai/pro (accessed on 15 February 2026)” were used exclusively for language refinement, limited to grammar, spelling, punctuation, and improving clarity of expression throughout the manuscript. The tool was not employed for generating data or performing statistical analysis. All AI-assisted text was critically reviewed, substantially edited, and approved by the authors to ensure scientific accuracy and integrity. No generative AI was used for study design, data collection, or interpretation. Prompts and outputs were not retained for public sharing; however, the authors confirm that all AI-generated content was thoroughly verified against primary sources and original research data.

## 4. Conclusions

This study demonstrated that an L-glutamate oxidase (EOx) can be rationally engineered into a virtually L-Glu dehydrogenase (*v*EDH) with minimal oxygen reactivity suitable for oxygen-independent electrochemical sensing of L-Glu, the primary excitatory neurotransmitter in the mammalian central nervous system. EOx from *Streptomyces mobaraensis* (SmEOx) was employed as it does not require post-translational modifications, making it more amenable to protein engineering. By identifying and mutating key residues (M117 and K400) involved in the oxidative half reaction with oxygen, we obtained the SmEOx variant M117F/K400N with markedly suppressed oxidase activity and greatly enhanced dye-mediated dehydrogenase activity, achieving a Dh/Ox ratio improved by ~6700-fold compared with the WT enzyme. Electrochemical evaluation using quasi-DET-dependent transient OCP measurements showed that the arPES-modified M117F/K400N *v*EDH enabled L-Glu detection under ambient oxygen without the need for external electron mediators, whereas the WT enzyme showed no response. These results not only overcame obstacles associated with oxygen dependence and engineering barriers imposed by post-translational requirements of existing enzyme-based L-Glu sensors but also established a foundation for developing miniaturized, real-time L-Glu monitoring systems with high temporal and spatial resolution.

## Figures and Tables

**Table 1:** Enzymatic activity properties of SmEOx WT and mutants.

	Dh Activity		Ox Activity		Dh/Ox [A]/[B] (-fold)
	*V*_max_ (U/mg)	*K*_m_ (mM)	*V*_max_ (U/mg)	*K*_m_ (mM)	
WT	0.28 *	n.c. *	93	3.0	0.0030
M117I	3.2	16	0.37 *	n.c. *	7.3
M117F	0.019 *	n.c. *	0.040	0.88	0.48
K400A	0.7	18	0.10	8.0	7.8
K400L	1.4	40	0.092	8.5	15
K400I	1.6	38	0.059	17	27
K400F	2.4	220	0.39	85	6.2
K400N	2.0	28	0.19	16	10
K400Y	0.8	67	0.054	15	15
M117I/K400I	1.5	65	0.062	35	24
M117F/K400I	0.3	1.8	n.d. **	n.d. **	n.c. *
M117F/K400N	3.5	63	0.18	25	20

[A]: *V*_max_ of dehydrogenase activity, [B]: *V*_max_ of oxidase activity. * n.c.: not calculated (The enzyme did not follow typical Michaelis–Menten kinetics, and *K*_m_ and *V*_max_ could not be reliably determined. For these mutants, the values shown in the *V*_max_ column represent the specific activity at the highest substrate concentration tested (100 mM L-Glu)). ** n.d.: not detected.

## Data Availability

The original contributions presented in this study are included in the article/Supplementary Materials. Further inquiries can be directed to the corresponding author.

## Supplementary Materials

The following supporting information can be downloaded at: https://www.mdpi.com/article/10.3390/ijms27062831/s1.
