# Supplementary material for: Creation of an Engineered Oxygen-Insensitive L-Glutamate Oxidase for the Application of Electrochemical L-Glutamate Sensors
Source: Int J Mol Sci. 2026 Mar 20;27(6):2831. doi: 10.3390/ijms27062831 (PMC13026155; doi:10.3390/ijms27062831)
Supplement: Supplementary file 1 [file ijms-27-02831-s001.zip › ijms-4091493-supplementary.pdf]

Creation of an Engineered Oxygen-Insensitive L-Glutamate Oxidase for the Application of Electrochemical L-Glutamate Sensors

Mika Hatada [1†], Shouhei Takamatsu [1,2 †], Ryutaro Asano [2], Kazunori Ikebukuro [2], Wakako Tsugawa [2] and Koji Sode [1,*]

[1] Lampe Joint Department of Biomedical Engineering, The University of North Carolina at Chapel Hill and North Carolina State University, Chapel Hill, NC 27599, USA; mika_hatada@med.unc.edu (MH)
[2] Department of Biotechnology and Life Science, Graduate School of Engineering, Tokyo University of Agri-culture and Technology, Tokyo 184-8588, Japan; ryutaroa@cc.tuat.ac.jp (RA); ikebu@cc.tuat.ac.jp (KI); tsugawa@cc.tuat.ac.jp (WT)

[†] These authors contributed equally to this work
* Correspondence: ksode@email.unc.edu

Supplementary Information

Supplemental Figure S1: Characterization of SmEOx mutants.
Supplemental Figure S2: SDS-PAGE analysis of SmEOx mutants purified by Ni-affinity chromatography.
Supplemental Figure S3: PMS concentration dependent dehydrogenase activity of wild type (WT) and K400F mutant SmEOx.
Supplemental Figure S4: Oxidase activity comparison between M117I and M117F/K400N mutants shown in the Figure 3(b) and (l).
Supplemental Figure S5: Cyclic voltammogram of arPES modified SmEOxs (WT, M117I, M117F/K400I, M117F/K400N) immobilized electrodes in 100 mM PPB pH6.0.
Supplemental Figure S6: Representative result of time dependent OCP change.
Supplemental Figure S7. Spectroscopic quantification of arPES modification on SmEOx (M117F/K400N).
Supplemental Figure S8. Position of Lys residues on the surface of SmEOx.

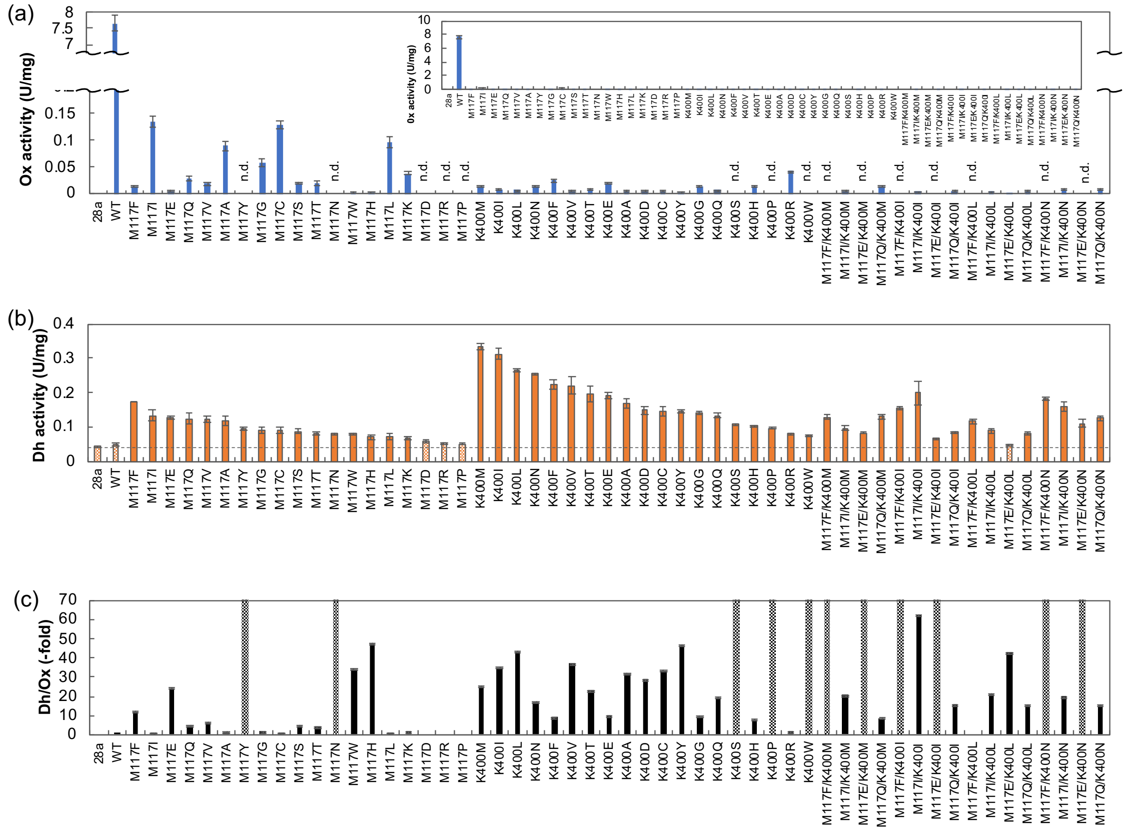

Figure S1. Characterization of SmEOx mutants.

(a) Oxidase (Ox) activity and (b) dye-mediated dehydrogenase (Dh) activity of mutant SmEOx against f.c. 100 mM L-Glu. (c) Ratio of Dh activity against Ox activity (Dh/Ox) of each mutant.

*n.d. : not detected

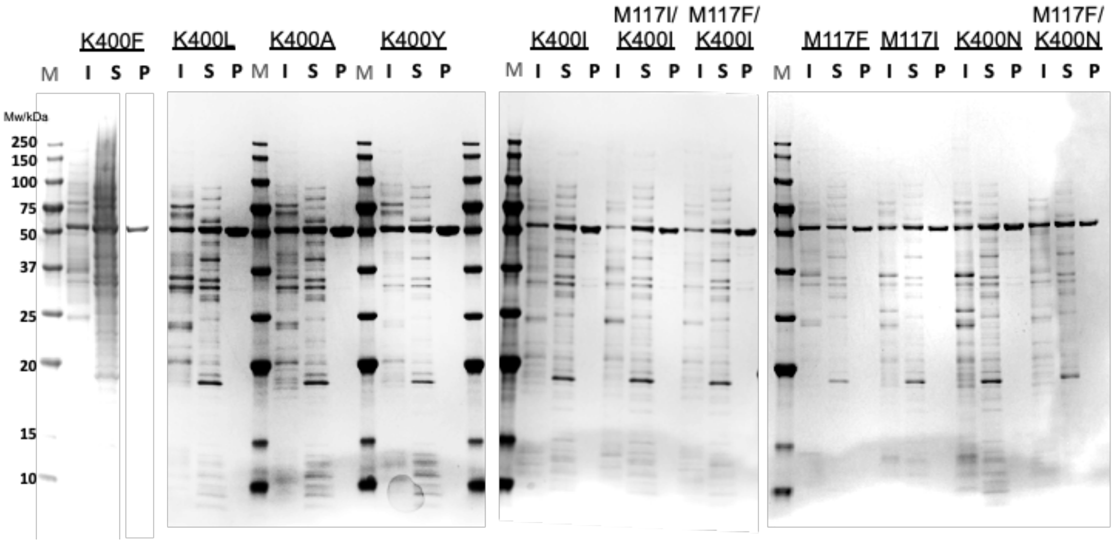

Figure S2. SDS-PAGE analysis of SmEOx mutants purified by Ni-affinity chromatography. Protein marker (M), insoluble fraction (I), soluble fraction (S) and purified sample (P) of SmEOx K400F, K400L, K400A, K400Y, K400I, M117I/K400I, M117F/K400I, M117F, M117I, K400N and M117F/K400N were analyzed.

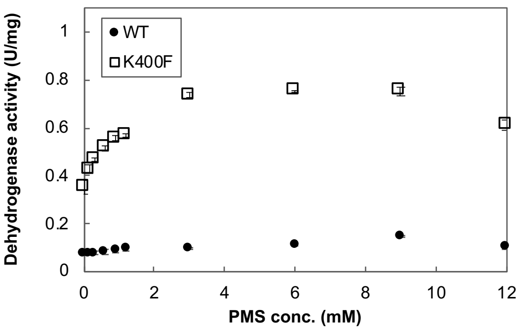

	$K_{m\ PMS}$ (mM)	V_{max} (U/mg)
WT	0.15	0.11
K400F	0.21	0.77

Figure S3. PMS concentration dependent dehydrogenase activity of wild type (WT) and K400F mutant SmEOx. Activity was measured in the presence of various concentrations of PMS (0, 0.15, 0.3, 0.6, 0.9, 1.2, 3.0, 6.0, 9.0, 12 mM), 0.06 mM DCIP and 100 mM L-Glu in 100 mM PPB pH 6.0 by monitoring the absorbance change at 600 nm, which associated with reduction of DCIP.

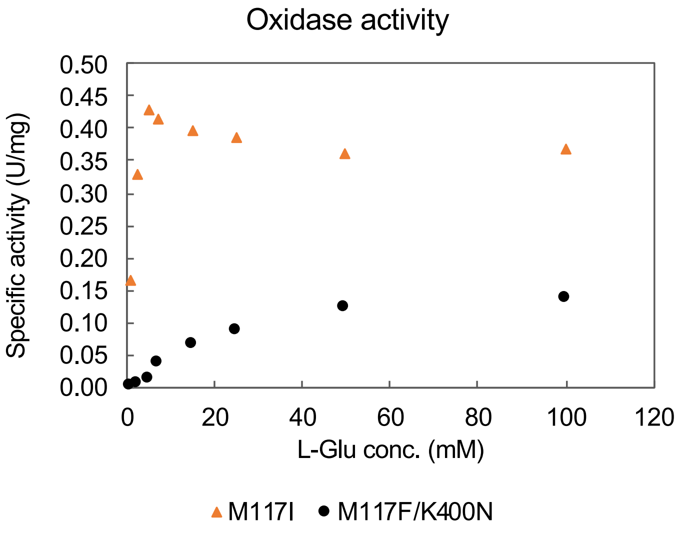

Figure S4. Oxidase activity comparison between M117I and M117F/K400N mutants shown in Figure 3(b) and (l).

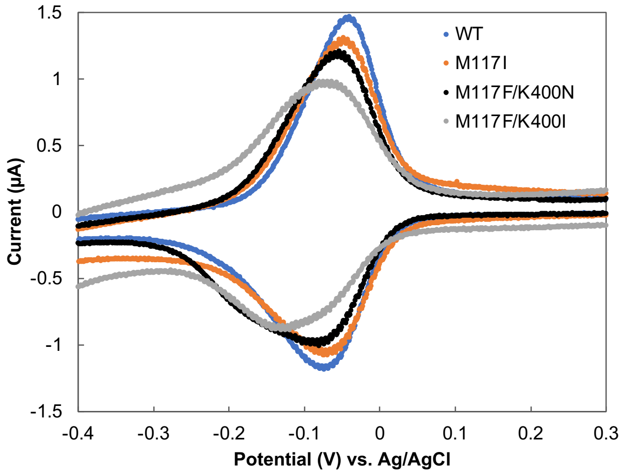

Figure S5. Cyclic voltammogram of arPES modified SmEOxs (WT, M117I, M117F/K400I, M117F/K400N) immobilized electrodes in 100 mM PPB pH 6.0.

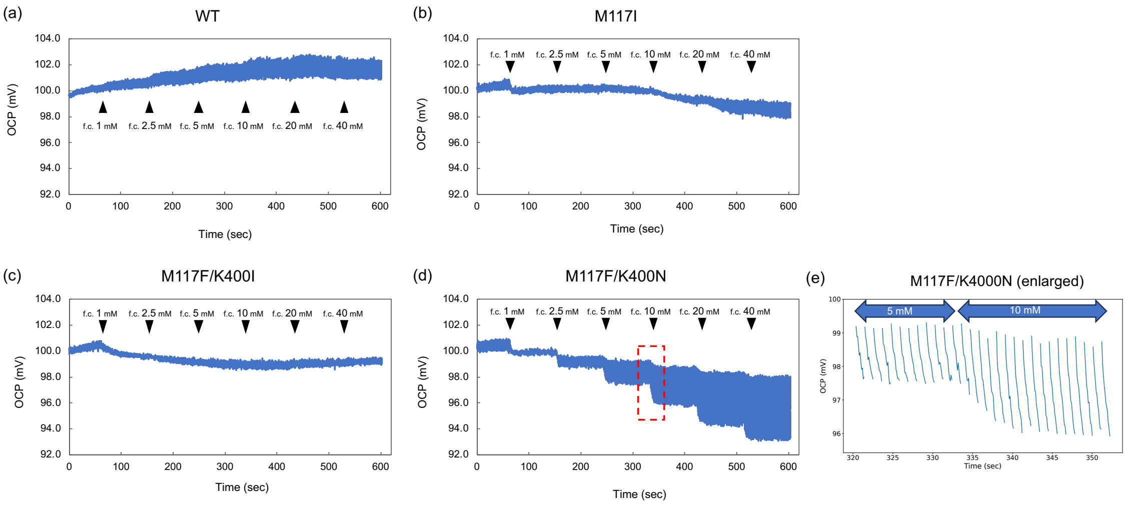

Figure S6. Representative result of time-dependent OCP change.

The raw data of OCP measurement using (a) WT, (b) M117I, (c) M117F/K400I, (d) M117F/K400N. An oxidizing potential of +0.1V vs. Ag/AgCl was applied for 0.1 sec followed by OCP measurement for 1 sec. This cycle was repeated continuously while the final concentration of L-Glu was increased by the addition of various concentration of L-Glu in the electrochemical test chamber. (e) The enlarged raw data of the OCP measurement corresponding to the area indicated by the red dashed square in (d).

The dOCP/dt was calculated for each 1 sec of OCP recording and plotted against the time to obtain the corresponding time-course shown in Figure 4(a) in the main text.

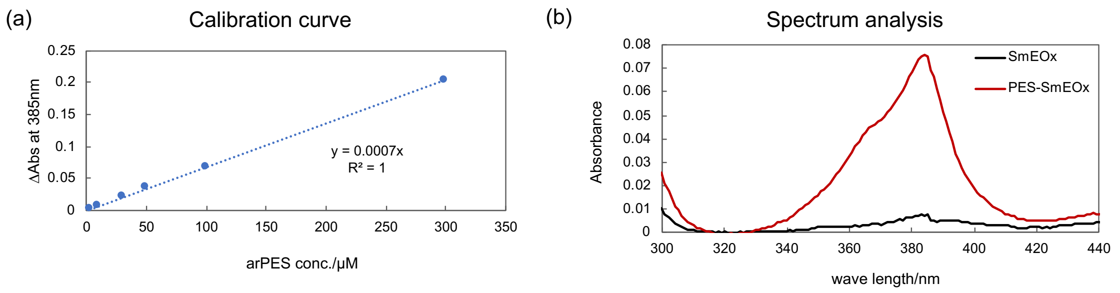

SmEOx concentration used for the spectrum analysis	10 μM
Calculated PES concentration of PES-SmEOx sample	96 μM
Calculated number of modified PES per enzyme	9.6

Figure S7. Spectroscopic quantification of arPES modification on SmEOx (M117F/K400N).

A mixture of 1 mg/mL (14 μM) SmEOx M117F/K400N and 1.7 mM arPES in 10 mM PPB (pH 6.0) was incubated at 4 °C overnight. Unbound arPES was removed by ultrafiltration. The absorbance at 385 nm, corresponding to PES, was measured to generate the calibration curve (a). The molecular extinction coefficient (ε) of PES calculated using this calibration curve was $\varepsilon = 1.4 \times 10^4\ M^{-1}cm^{-1}$.

Spectral analysis was then performed using 10 μM unmodified and PES-modified SmEOx (b).

Each absorbance spectrum was normalized using the absorbance value at 315 nm. The difference in absorbance at 385 nm between unmodified and PES-modified SmEOx was used to calculate the PES concentration in the PES-modified enzyme using the calibration curve. The calculated PES concentration and the corresponding number of PES molecules attached per enzyme are shown in the table.

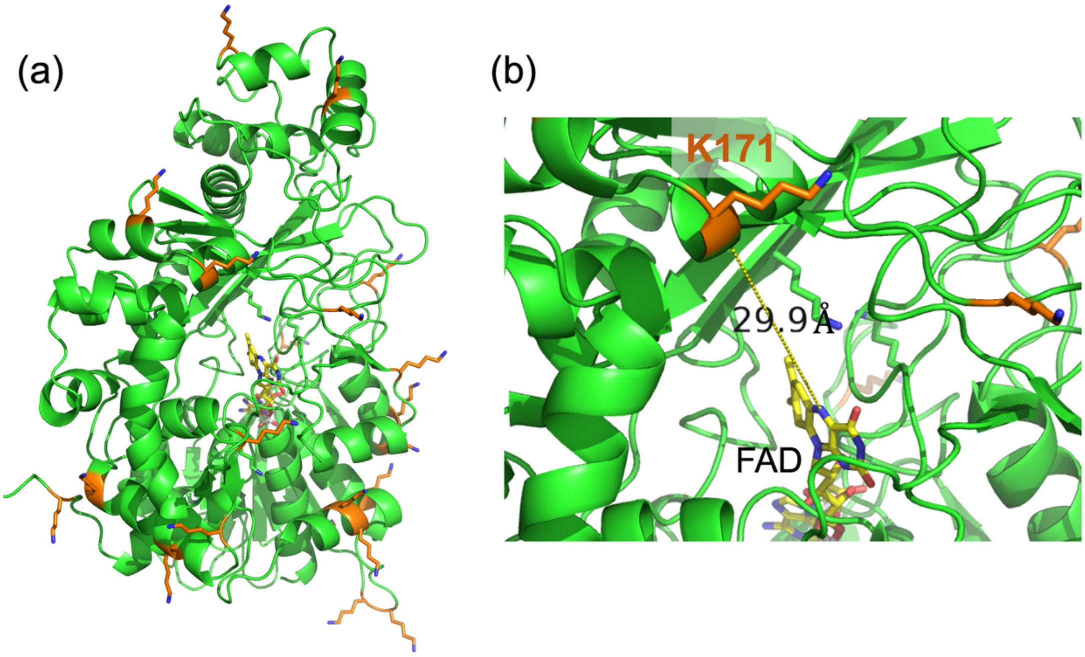

Figure S8. Position of Lys residues on the surface of SmEOx. The Lys residues located on the surface of SmEOx are shown as orange sticks: (a) A total of 21 Lys residues are located on the surface of SmEOx. (b) The distance between the N5 atom of FAD and the nearest surface Lys residue (K171) is 29.9 Å.
